# Natural Flora Is Indiscriminately Hosting High Loads of Generalist Fungal Pathogen *Colletotrichum gloeosporioides* Complex over Forest Niches, Vegetation Strata and Elevation Gradient

**DOI:** 10.3390/jof9030296

**Published:** 2023-02-24

**Authors:** Pauline Dentika, Margot Gumbau, Harry Ozier-Lafontaine, Laurent Penet

**Affiliations:** Institut National de Recherche Pour L’Agriculture, L’Alimentation et L’Environnement (INRAE), Research Unit ASTRO, F-97170 Petit-Bourg, Guadeloupe, France

**Keywords:** *Colletotrichum gloeosporioides* species complex, anthracnose risk, natural vegetation, vegetation strata, fungal niche

## Abstract

Crop pathogenic fungi may originate from reservoir pools including wild vegetation surrounding fields, and it is thus important to characterize any potential source of pathogens. We therefore investigated natural vegetation’s potential for hosting a widespread pathogenic group, *Colletotrichum gloeosporioides* species complex. We stratified sampling in different forest environments and natural vegetation strata to determine whether the fungi were found preferentially in specific niches and areas. We found that the fungi complex was fairly broadly distributed in the wild flora, with high prevalence in every study environment and stratum. Some significant variation in prevalence nevertheless occurred and was possibly associated with fungal growth conditions (more humid areas had greater prevalence levels while drier places had slightly lower presence). Results also highlighted potential differences in disease effects of strains between strata components of study flora, suggesting that while natural vegetation is a highly probable source of inoculums for local crops nearby, differences in aggressiveness between vegetation strata might also lead to differential impact on cultivated crops.

## 1. Introduction

Plant diseases are a serious factor in limiting crop production, and pathogens may attack cultivated plants at all stages of their life cycle [1], from germination to senescence, including post-harvest storage of foods [2]. Diseases take advantage of genetically homogenous fields at large scales, seed chain contaminations [3], and are explosive when favourable weather conditions for epidemics are met [4]. Nevertheless, many diseases are constrained by specificity in host range and specialisation due to their co-evolutionary nature [5], so that control can be somewhat efficient with proper regional monitoring effort, varietal turn-over [6] or multiline varietal strategies, and appropriately managed biocides [7]. On the other hand, disease control will be much harder for diseases resulting from more generalist pathogens—especially fungi, with epidemic bursts sometimes more difficult to anticipate [8].

While epidemic bursts are the result of favourable circumstances such as genetic homogeneity of cultivated varieties at broad scale [9] and weather conditions conducive to both explosive multiplication and dispersal (either passively from winds and rains [10] or more actively via vectors), inoculum sources play a major role in disease initiation, especially in proximity of fields [11,12,13]. As such, origin of inocula is a major focus of research in plant pathology [14], along with monitoring disease risk and spread over regions [15]. Specialist pathogens will have a narrower range of favourable circumstances for epidemics [16], possibly correlating more strongly to agronomic practices [17,18] and flow in the production chain (including storage and seeds distribution, see [2]) and are thus more amenable to control. On the other hand, generalist pathogens will have a broader spectrum of favourable circumstances, including ecological interactions resulting in disease initiation [19]. For generalist fungi, a specific issue is that of host plants allowing pathogens to survive intercrop season and initiate new epidemics [11], and sometimes proximal field vegetation is directly an inoculums source [12,13]. Therefore, deciphering host range and natural ecology of fungi is especially relevant to understanding plant disease risk [19,20,21], especially *Colletotrichum* species in their natural environment [22].

In this study, we investigated the potential role of natural flora in hosting potentially pathogenic species from the *C.gloeosporioides* complex. The fungal complex is indeed responsible for initiating anthracnose disease on water yams (*Dioscorea alata*) [21] in the Caribbean [17,23], despite breeding efforts against the disease [24] and a diverse pool of progenitors [25]. Anthracnose disease at epidemic stages often results in widely necrotic plants unable to sustain photosynthesis anymore and sometimes leads to complete crop wipeout and dramatic harvest losses [26]. Yet, little is known about inoculum sources. Seed tubers were once hypothesized as a plausible origin [27], since the fungus produces skin disease on tubers [28,29] and might start disease in young plants. The species complex is known to infect numerous plant hosts [30,31,32], even in natural settings, though despite a potentially widespread range it is also known to behave nearly symbiotically at times [33], sometimes even turning saprophytic [34,35]. Reasons why strains become harmful to crops are not yet fully understood, though the nature of species complex might play a role [36]. Recent studies have shown that weed species host *C. gloeosporioides* and may behave as inoculums’ relay during intercropping [11]), as well as field hedges [12,13]. In light of these results, we investigated the prevalence of the fungi in natural vegetation, as several studies have documented broad prevalence range in the wild [37] and local abundance in the study region [13], a situation often encountered with fungi in general [38]. We decided to closely focus on vegetation strata along an elevation gradient in a natural secondary growth forest in order to test whether host skill differed not among species but among ecological niches and vegetation strata, thus adding to current knowledge of *Colleotrichum* presence in woody vegetation [22]. We thus asked the following questions: What is the prevalence of the pathogen in natural vegetation? Does it vary across strata (canopy, understory, and floor)? Is it sensitive to elevation or changes in relative humidity or wind levels?

## 2. Materials and Methods

**Figure 1:**
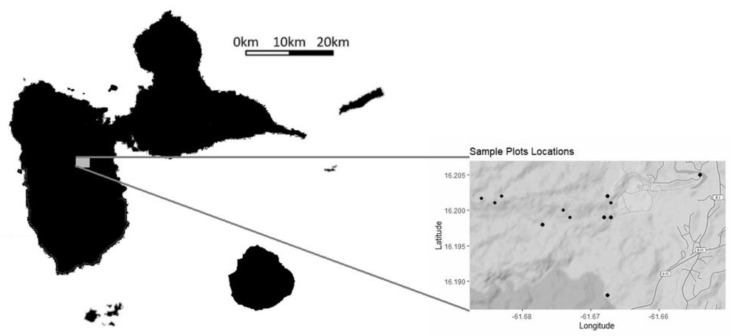
Study area. Study plots were sampled in the island of Guadeloupe.

Sample collection and study site. In January and February 2019, sampling sessions were conducted at various locations within a naturally regenerating secondary tropical forest a few hundred meters from yam fields in the Basse-Terre region of the Caribbean island of Guadeloupe. Plots were chosen randomly along an elevation gradient (from river to hilltop) and a total of 12 locations were sampled. Plot locations are the following: 16°11′52″ N 61°40′37″ W, 16°11′57″ N 61°40′03″ W, 16°11′57″ N 61°40′04″ W, 16°11′58″ N 61°40′03″ W, 16°11′58″ N 61°40′25″ W, 16°11′59″ N 61°40′25″ W, 16°12′06″ N 61°40′03″ W—for 2 nearby plots, 16°12′06″ N 61°41′03″ W—for 3 nearby plots, and 16°12′18″ N 61°39′13″ W. Elevation above sea level (m) was recorded for each plot (Figure 1). These locations were stratified so as to account for 4 different conditions of the vegetation: ‘riparian forest’ (within twenty meters of the river of Bras David, i.e., very humid conditions), ‘deep forest’ (without specific conditions beyond distance to next walking path > 15 m), ‘forest edge’ (within ten meters of the forest edge, i.e., a more open environment), and ‘hill forest’ (atop of hill, distance to next walking path > 15 m). For each plot, vegetation was sampled for all three strata defined as follows: ‘floor’, i.e., small plants species below 20 cm in size; ‘understory’, i.e., tall herbs and shrubs with sizes greater than 50 cm above the soil; and ‘canopy’, i.e., tree species, though sampling was done at the lowest height (ca. 2 to 5 m above the soil).

For each of these strata, 10 plants were sampled for leaves and pictured for further identification, thus yielding 30 samples per plot, and thus a grand total of 12 × 30 = 360 plant sampled. For each sample plant, a leaf in perfect condition (‘healthy’, i.e., without external sign of disease) was sampled, and whenever possible, another leaf with potential disease symptoms was sampled too (‘diseased’, i.e., with apparent necrotic spots), with symptoms usually associated with fungal disease in general (necrotic spots, either dry, typical of a hypersensitive reaction in a gene for gene interaction, or wet rot, or larger aggregating spots, etc.). Leaves were picked directly from the sample plants and immediately placed in hermetic plastic bags labelled with a sample code. All sample bags were left in a refrigerated cooler box until field work was completed. Plant species identification was further processed with the help of Fournet Flora [39] and colleagues with expertise on local vegetation. Every study species had at least one individual hosting a strain of *C. gloeosporioides* complex (the list of study species can be found in the opening of the Section 3).

**Figure 2:**
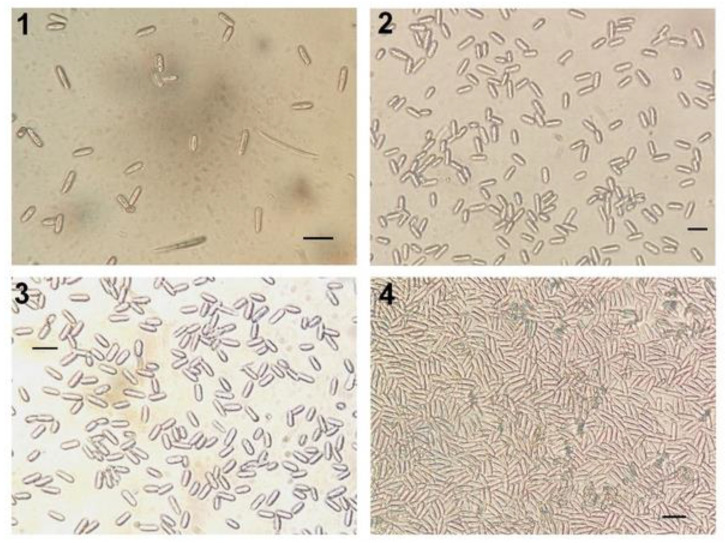
Some isolated strains. Scale bars are 20 µm. Strain from (**1**) a leaf with typical fungal necrosis from *Ipomea setifera* (floor strata in edge environment, ‘diseased’ leaf), (**2**) a diseased leaf from *Phyllanthus mimosoïdes* tree (canopy strata deep forest environment), (**3**) a healthy leaf from *Philodendron lingulatum* (understory strata from riparian environment), and (**4**) a healthy leaf from *Psychotria urbaniana* (understory strata from hill environment).

Specimen culture and examination. Cooler boxes were brought back to the lab in the afternoon to allow isolation of fungal strains from sampled leaves. Leaves were first washed in successive baths during about half a minute, first in a 10% diluted bleach solution, then rinsed in distilled water, then a short 70% methanol bath, and one last rinsing phase in water. Further isolation was performed in sterile conditions under a laminar flow cabinet (model LRF 48). Leaf pieces were cut and placed on Petri dishes with S medium (see [11,12]) to increase odds of sampling *Colletotrichum* species, which were further sealed with parafilm tape according to routine lab procedures. After an incubation time of 4 to 6 days under 12 h light (under Osram T8 L 36 W/865 Lumilux DaylightG13 neons, similar to daylight) at room temperature (22–28 °C), conidia from the Petri dishes were observed under a light microscope for species complex identification based on spore morphology [40] (Figure 2) and to estimate prevalence of species from the *C. gloeosporioides* complex from sampled leaves. Continuous hyaline conidia with quite regular, cylindric, straight shape and ends rounded of about 20 µm were assigned to our focus strains [41]. (Please note that a previous study of ca. 550 strains sampled on *D. alata* yams in the island selected with the same criteria yielded 100% assignment to *C. gloeosporioides* complex using ITS probe—Dentika et al. in prep).

Our strategy in this study was to use a morphospecies concept and derive general knowledge of *C. gloeosporioides* as a species complex, rather than focusing on sequence-based taxonomy as currently highlighted for the complex [31]. This complex accounts for a probable worldwide two hundred species or more if analysed via species assignment with sequence analysis [30]. As a result, working at a morphospecies level will be less precise and lump information for several systematic entities. Nevertheless, closely related fungi are known for fairly fuzzy species delineation and potent gene admixing and recombination [42]. All of our samples coexisted locally next to each other and are necessarily a reduced subset of total entities from the global species complex. Preliminary analyses of local species have shown that local members of *C. gloeosporioides* complex in Guadeloupe segregate similarly among *C. alatae*, *C. siamense*, and *C. fructicola* species (unpublished results). While some variation might exist as to the exact host range of sample entities, or the probability of gene exchange between them, the ubiquity of the complex locally makes our study a fair assessment for deriving generalities and disease risk for neighbouring crops.

Statistical analyses. Data were organised following their natural order: environment (riparian, deep, edge, hill), stratum (floor, understory, canopy), altitude (in m), and recorded presence of a strain belonging to *C. gloeosporioides* complex from either a symptomatic leaf (‘diseased’, encoded 0 or 1 when a strain is present), a leaf without symptoms (‘healthy’, encoded 0 or 1 when a strain is present), or present in either plant leaf (‘global’, encoded 0 or 1 when present). Samples were the following: 222 leaves with symptoms and 350 asymptomatic leaves (some sampled plants had only leaves presenting symptoms, while most had only asymptomatic leaves). We first produced logistic regression models in R, investigating either prevalence (diseased, healthy, global) as dependent factors, with environment and stratum as independents, altitude as a covariate, and their interactions (two levels and three levels) in a full factorial analysis. This helped us detect factors with impacts on prevalence levels of the fungi within natural vegetation. In a second step, we estimated local prevalence (plot level) for each diagnosis (diseased, healthy, global) for every stratum and produced a complete correlogram to estimate relationships between prevalence at each factor level (for example, correlations between strata, or between diseased and healthy estimates). This allowed us to discuss the relationship between prevalence and strata in light of the potential contamination chain (either active via winds or passive between strata within plots via drops falling from higher strata during rains). All analyses were conducted with R software (version 4.1.0) [43].

## 3. Results

### 3.1. General Comment

Overall prevalence of species from *C. gloeosporioides* complex was important in natural vegetation: 0.689 (153 strains out of 222 leaves) for diseased sample category, 0.717 (251 strains out of 350 leaves) for healthy sample category, with a global prevalence of 0.805 (290 plants out of 360 samples). In this study, we isolated strains from *C. gloeosporioides* complex at least once (and up to nine times) in the 71 following plant species sampled in the wild, which should thus be considered potential hosts for the fungi: *Abarema jupunba*, *Anemia adiatifolia*, *Anthurium palmathum*, *Asplundia *sp., *Axonopus equitens*, *Axonopus *sp., *Bidens alba*, *Brachiaria *sp., *Calopogonium mucunoïdes*, *Centella asiatica*, *Centrosoma pubescens*, *Clidemia hirta*, *Coccoloba *sp., *Commelina difusa*, *Cyathea *sp., *Desmodium axilare*, *Desmodium barbatum*, *Desmodium incanum*, *Desmodium *sp., *Desmodium trifolium*, *Dicranopteris linearis*, *Dieffenbachia *sp., *Elephantopus mollis*, *Epipremnum aureum*, *Eugenia *sp., *Geophila repens*, *Gonzalagunia spicata*, *Heliconia *sp., *Hyptis atrorubens*, *Hyptis *sp., *Ichnanthus pallens*, *Inga ingoïdes*, *Ipomea setifera*, *Ipomea tilliacea*, *Licania ternatensis*, *Miconia mirabilis*, *Mimosa pigra*, *Mitracarpus hirtus*, *Myrcia fallax*, *Myrcia *sp., *Pachira aquatica*, *Panicum tricoïdes*, *Philodendron gigantea*, *Philodendron lingulatum*, *Phyllanthus mimosoïdes*, *Piper dilatatum*, *Polypodium chelypteris*, *Psychotria urbaniana*, *Pueraria phaseloides*, *Richeria grandis*, *Rolandra fructicosa*, *Sarcorhachis incurva*, *Sauvagesia erecta*, *Scindapsus *sp., *Scleria secans*, *Selaginela flabellata*, *Simareouba amara*, *Sloanea *sp., *Solanum torvum*, *Spathoglottis plicata*, *Spermacoce *sp., *Spermacoce assurgens*, *Stachytarpheta jamaicensis*, *Sterculia caribaea*, *Sterculia *sp., *Stylogyne triflora*, *Switenia marogani*, *Syzygium jambos*, *Syzygium *sp., *Teramnus labialis*, *Tilesia baccata*, and *Wedelia trilobata*. A strain was isolated from every sampled species at least once in this study, though not every sampled plant was contaminated.

### 3.2. Effect of Environment, Stratum, and Altitude on Colletotrichum Prevalence

**Figure 3:**
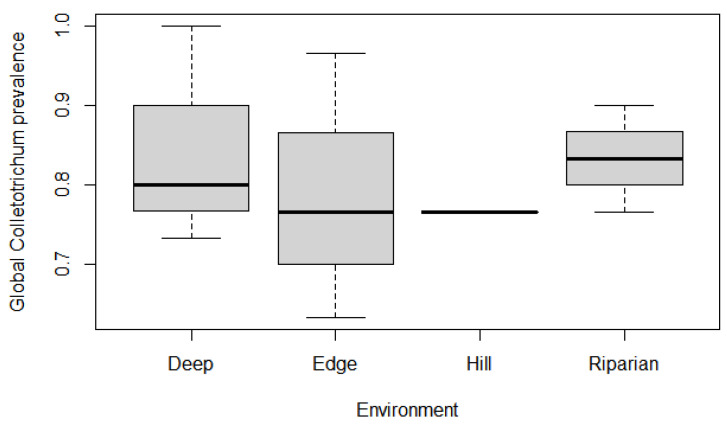
Global prevalence of species from *C. gloeosporioides* complex in the different forest environments. Hill forest plots had marginally lower prevalence (*p* < 0.1), mostly due to fairly lower variation in prevalence compared to other niches, and riparian forest plots had significantly greater prevalence (*p* < 0.05). Each environment is estimated via three plots.

**Figure 4:**
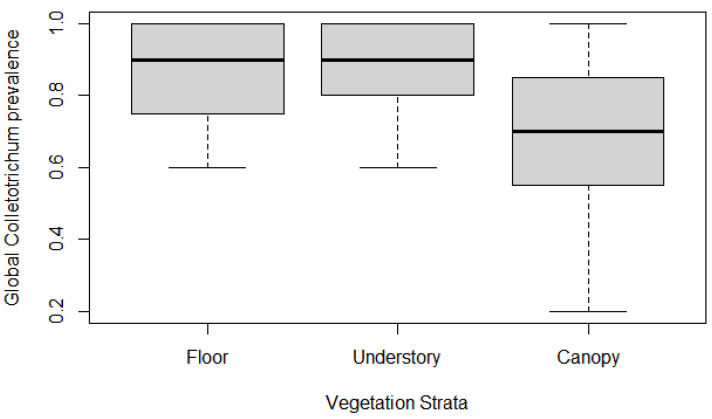
Global prevalence of species from *C. gloeosporioides* complex in the different vegetation strata. ‘Floor’ and ‘understory’ have slightly greater and marginally significant prevalence (*p* < 0.1) compared to ‘canopy.’ Each stratum is estimated via 12 plots.

**Figure 5:**
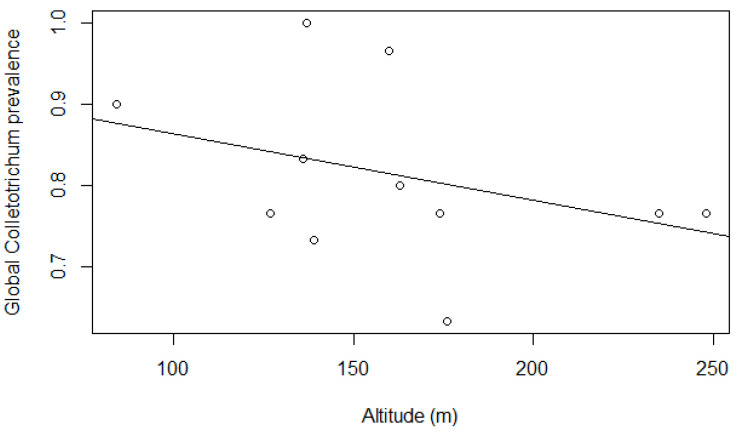
Effect of altitude on global *Colletotrichum* prevalence (*p* < 0.0345, Table 1). For the sake of clarity, the average for each spot is reported, though data are binomial at plant level.

Overall, *Colletotrichum* prevalence was high in every environment and stratum, though for the global prevalence model, some conditions differed from others (Table 1): *Colletotrichum* prevalence in riparian forest was significantly greater than baseline (‘deep forest’) (*p* = 0.0415), and prevalence in hill forest was marginally significantly lower (*p* = 0.099) (Figure 3). Both ‘floor’ and ‘understory’ strata had marginally significant prevalence greater than baseline (canopy) (*p* = 0.0977 and 0.0585 respectively) (Figure 4). Altitude significantly impacted prevalence (*p* = 0.0348), with a decrease in fungi presence in vegetation as elevation increased (Figure 5). Few interaction terms were significant in the model, but prevalence of *Colletotrichum* in ‘understory’ strata in ‘riparian forest’ was much greater (*p* = 0.0394) and altitude effect of decreasing prevalence was more pronounced in ‘hill Forest’ ‘understory’ (*p* = 0.0351) (Table 1).

Several interaction terms were marginally significant and indicative of a pattern of increased prevalence (e.g., ‘floor’ strata in ‘riparian Forest’, ‘understory’ strata in ‘hill forest’), and altitude demonstrated a pattern of decreasing prevalence more markedly in ‘hill’ and ‘riparian’ forest, and in ‘floor’ and ‘understory’ strata. Models with healthy and diseased leaves similarly had high levels of prevalence in either condition, though none of them differed significantly from the others and will thus not be further discussed.

### 3.3. Correlations among Colletotrichum Prevalence and Infection Dynamics in Vegetation Strata

**Figure 6:**
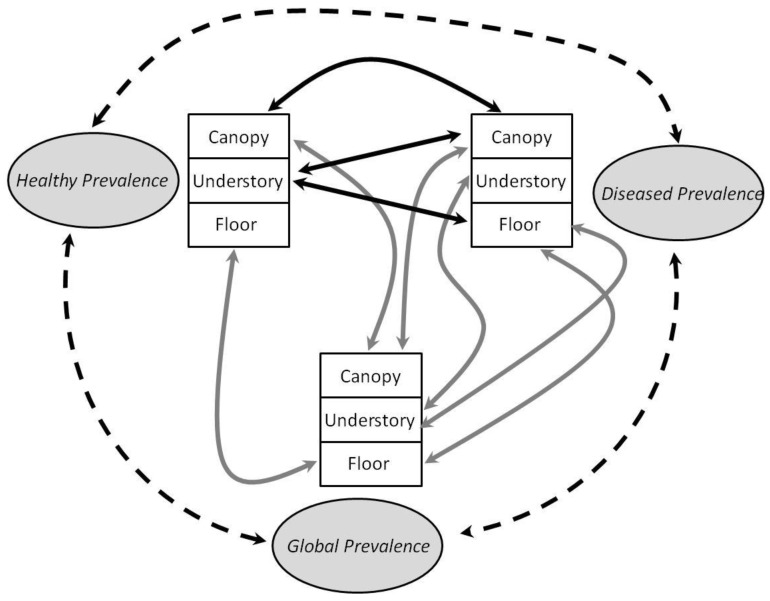
Scheme of significant correlations for all *Colletotrichum* prevalence, at global and strata levels. Only significant correlations are illustrated. Dash lines associate plot prevalence; grey lines associate strata prevalence from either diseased or healthy leaves estimates to their global equivalent; and black lines associate diseased or healthy estimates to their counterpart correlate. All correlations were positive. Coefficient values are reported in Table 2.

Most prevalence estimates (‘diseased’ or ‘healthy’, see Table 2 for details) correlated significantly to ‘global’ estimates by strata (e.g., ‘diseased canopy’ correlated to ‘global canopy’, see Figure 6), save ‘healthy understory’, which was not correlated to ‘global understory.’ Inter-conditions (significant ‘diseased’ to ‘healthy’ correlations) were few: ‘healthy canopy’ prevalence correlated to ‘diseased canopy’ prevalence, and ‘healthy understory’ prevalence correlated to both ‘diseased canopy’ and ‘diseased floor’ prevalence. These patterns can be interpreted in asymmetrical contributions of the different strata in infection dynamics and potential strain aggressiveness (see Section 4).

## 4. Discussion

*C. gloeosporioides* complex is considered an ubiquitous worldwide species and our results confirmed a widespread presence of these crop pathogen fungi in natural forest vegetation, generally at fairly high prevalence (average: 0.71; range 0.33–1.00), independent of environmental niche or vegetation strata, to the exception of places where conditions for growth were impacted (e.g., more humid riparian forest plots had greater prevalence, drier hill plots had slightly lower prevalence, and altitude generally decreased presence of the fungi). Patterns of correlations between prevalence in the different conditions (diseased or healthy leaves) and vegetation strata indicated differential influence on infection dynamics: healthy canopy prevalence was closely associated with diseased canopy prevalence, possibly suggesting a first filtering effect in canopy within a diverse pool from spore rain, and increased prevalence led to increased disease levels. However, if higher disease levels in canopy were expectedly associated with higher disease levels in floor strata, they were also strikingly correlated to healthy prevalence in understory. This result suggested that strains differentially affect plant species within different strata. We will discuss these findings within the guiding principle of potential impact on cultivated fields and crops.

*Colletotrichum* is a generalist fungus, historically thought of as involving specialist relationships with very narrow host range (i.e., following single interactions pairs—a pathogenic species associated to a plant species), sometimes inducing taxonomic confusions [31], then transiently interpreted via the length of morphospecies complexes [40], but today interpreted as having broad host species range within species complexes [30]. Indeed, we describe here a fairly wide array of host species coexisting locally and most probably with an important share of strains. Prevalence was high in our population sample (a known feature of the complex [37]), and this was true within every forest niche and vegetation stratum. It was indeed even higher in natural vegetation than it was in weeds communities found in fields from very close (1 km) to regional distance (within 20 km) [11]. Perhaps most importantly, among the 71 plant species hosting *Colletotrichum* fungi (listed above), at least 27 are commonly found here and there in field edges or even within fields (e.g., *Bidens alba*, *Calopogonium mucunoïdes*, *Centella asiatica*, *Centrosoma pubescens*, *Clidemia hirta*, *Commelina difusa*, *Cyathea* sp., *Desmodium axilare*, *Desmodium barbatum*, *Desmodium incanum*, *Desmodium* sp., *Desmodium trifolium*, *Elephantopus mollis*, *Heliconia* sp., *Hyptis atrorubens*, *Hyptis* sp., *Inga ingoïdes*, *Ipomea setifera*, *Ipomea tilliacea*, *Miconia mirabilis*, *Mimosa pigra*, *Mitracarpus hirtus*, *Stachytarpheta jamaicensis*, *Solanum torvum*, *Spathoglottis plicata*, *Stachytarpheta jamaicensis*, *Syzygium jambos*, and *Wedelia trilobata*), and some of them are already known hosts to *C. gloeosporioides* complex [11]. Amazingly, there seems to be a continuum in prevalence from broadly inoculated natural vegetation to cultivated areas where fungi presence is much scarcer (weed communities around fields and monocultural crop themselves, even susceptible species), suggesting *Colletotrichum* might best be seen as conquering agricultural land, and possibly in that process producing disease in crops. This idea might also explain why the fungi occur as such extremes as peaceful leaf commensal [33,37] or as strongly pathogenic and driving anthracnose disease in crops [44]. A consequence of this is that some local farmers shifted species cultivation in order to reduce disease impact on yams [18].

Despite high prevalence in general, our results also highlighted that some conditions might be limiting or on the contrary conducive to propagation. Indeed, riparian forest had higher rates of fungus presence, especially for understory and floor strata (Table 1), and this might reflect more humid conditions favourable to fungus growth. On the other hand, top hill forest plots were places of lower prevalence (Figure 3), and altitude was consistently associated with a weaker presence of *Colletotrichum* (Figure 4). Of course, these are areas associated with drier atmospheric conditions, though these are also more exposed to winds, which is an important factor in spore dispersal and thus arrival of the fungi as well. The evidence would thus point to local conditions for installment and growth being more restrictive in explaining fungus prevalence than long distance dispersal (but see [45]). The pattern of lower prevalence at forest edges with greater variance (thus making edge statistically no different than deep forest) seems to corroborate this observation further. In addition, the pattern of increasing prevalence between canopy and understory or floor obviously reinforces the idea that local inoculation is rather passive through rains once *Colletotrichum* species successfully installs in canopy and that more humid conditions such as those expected below a canopy will increase odds for the fungus to inoculate other plant species, a situation similarly documented in field crops [46]. Local conditions will thus allow for higher presence of *Colletotrichum* if they are favourable to fungal growth compared to other locations undergoing a more important spore rain but drier and harsher growth conditions.

Local inoculation dynamics are thus dependent on growth conditions, but our results suggested that other processes were at play, and that strata may respond differently, especially regarding disease status (prevalence from diseased leaves vs. from healthy leaves). Indeed, prevalence estimates were sometimes correlated in unexpected ways: for example, diseased canopy prevalence was strongly correlated to diseased floor prevalence but also very strongly to healthy understory prevalence. These correlations demonstrate that strains producing disease in canopy may well also be aggressive in lower strata too (e.g., floor species), but apparently do not necessarily translate into disease for understory species. It is unclear why such a pattern emerged, though possible hypotheses might focus either on species effect or possible specific ecophysiological features in the different strata (e.g., cuticle thickness and composition). If such effects were replicated, it would be of interest to investigate whether strain aggressiveness differential impacts risk of disease such as anthracnose when strains escape natural vegetation and disperse into cultivated areas (see discussion in [11]). This would explain why so many *Colleotrichum* strains seldom produce disease in crops known to be sensitive, while difficult-to-predict epidemics can suddenly put specific crops at risk when aggressive strains land in the right place.

Natural vegetation is thus an important reservoir of potentially pathogenic strains of species from *Colletotrichum gloeosporioides* complex, given the broad host range exhibited. On the other hand, these results are most plausibly true for the other *Colletotrichum* complexes [47,48], and possibly other fungi with broad host range and affinity to crops. Understanding fungal dynamics and how they translate into increasing disease risk for crops is a pressing issue in the wake of agriculture transition toward reduced use of synthetic inputs, as fungi propagate near and within fields [11,12,13]. Disease control might nevertheless benefit from an extended microbiome approach in agriculture, for which pathogen displacement may be reached under field ecological conditions, provided microbial community functioning is better described [49]. Indeed, interactions between fungi are known to lead to competition and negative interactions, including for *Colletotrichum* complexes where apparent antinomy within weeds between members of *C. acutatum* complex and *C. gloeosporioides* [11] impacted anthracnose development and reduced disease symptoms in yams [50]. Another approach relying on endosymbiotic relationships may also provide opportunities for disease control [51].

## 5. Conclusions

In summary, we described a broad presence of species from *C. gloeosporioides* complex in natural vegetation, and high prevalence was a feature of every niche and vegetation strata investigated. There were some effects attributable to local conditions, especially those associated with humidity and dryness, known to positively and negatively impact fungal growth, respectively. More humid environments might indeed be more prone to hosting important and diverse populations of the pathogen. We also note that prevalence was greater in natural vegetation than cultivated settings and the local flora and environment might well be considered important sources of diverse inocula for crops. Filtering effects of strain pools are nevertheless at play and may have quite different impacts on disease development depending on their origin, especially regarding strata. These filter effects are an important component in the study of epidemics and should be the focus of further research.

## Figures and Tables

**Table 1:** Effects of environment, vegetation strata, and their interactions in the global prevalence of species from *C. gloeosporioides* complex in natural forest vegetation. Factors with significant z-values are indicated in bold (and marked with an *), while factors with marginally significant z-values are indicated in italics.

Logistic Regression Model	Estimate	Std. Error	z Value	Pr(>|z|)
**(Intercept)**	**1.733 × 10^1^**	**7.704 × 10^0^**	**2.249**	0.0245 *
Environment-Edge	1.037 × 10^2^	8.743 × 10^1^	1.186	0.2357
*Environment-Hill*	*−3.058* *× 10^1^*	*1.854* *× 10^1^*	*−1.650*	*0.0990*
**Environment-Riparian**	**−1.625** **× 10^1^**	**7.970** **× 10^0^**	**−2.039**	0.0415 *
*Stratum-Floor*	*−1.713* *× 10^1^*	*1.034* *× 10^1^*	*−1.656*	*0.0977*
*Stratum-Understory*	*−1.914* *× 10^1^*	*1.012* *× 10^1^*	*−1.892*	*0.0585*
**Altitude**	**−1.039** **× 10^−1^**	**4.922** **× 10^−2^**	**−2.111**	0.0348 *
Environment-Edge. Stratum-Floor	1.265 × 10^3^	6.116 × 10^4^	0.021	0.9835
Environment-Hill. Stratum-Floor	3.176 × 10^1^	2.663 × 10^1^	1.193	0.2330
*Environment-Riparian. Stratum-Floor*	*3.141* *× 10^1^*	*1.869* *× 10^1^*	*1.681*	*0.0928*
Environment-Edge. Stratum-Understory	−1.021 × 10^2^	8.881 × 10^1^	−1.149	0.2504
*Environment-Hill. Stratum-Understory*	*5.017* *× 10^1^*	*2.617* *× 10^1^*	*1.917*	*0.0552*
**Environment-Riparian. Stratum-Understory**	**2.456** **× 10^1^**	**1.193** **× 10^1^**	**2.060**	0.0394 *
Environment-Edge. Altitude	−5.915 × 10^−1^	5.007 × 10^−1^	−1.181	0.2375
*Environment-Hill. Altitude*	*1.629* *× 10^−1^*	*8.615* *× 10^−2^*	*1.891*	*0.0586*
*Environment-Riparian. Altitude*	*1.006* *× 10^−1^*	*5.215* *× 10^−2^*	*1.929*	*0.0538*
*Stratum-Floor. Altitude*	*1.154* *× 10^−1^*	*6.830* *× 10^−2^*	*1.689*	*0.0912*
*Stratum-Understory. Altitude*	*1.275* *× 10^−1^*	*6.693* *× 10^−2^*	*1.905*	*0.0568*
Environment-Edge. Stratum-Floor. Altitude	−7.192 × 10^0^	3.475 × 10^2^	−0.021	0.9835
Environment-Hill. Stratum-Floor. Altitude	−1.744 × 10^−1^	1.233 × 10^−1^	−1.414	0.1574
Environment-Riparian. Stratum-Floor. Altitude	−2.151 × 10^−1^	1.359 × 10^−1^	−1.583	0.1135
Environment-Edge. Stratum-Understory. Altitude	5.821 × 10^−1^	5.097 × 10^−1^	1.142	0.2533
**Environment-Hill. Stratum-Understory. Altitude**	**−2.548** **× 10^−1^**	**1.209** **× 10^−1^**	**−2.107**	0.0351 *
*Environment-Riparian. Stratum-Understory. Altitude*	*−1.559* *× 10^−1^*	*8.349* *× 10^−2^*	*−1.867*	*0.0619*

**Table 2:** Correlation between prevalence estimates. In dark, Pearson correlation values between general estimates (healthy, diseased, global), and within strata estimates for each estimate (significant correlations were used to produce Figure 6). In grey, Pearson correlation values between general estimates and subsequent strata estimates for each category. Asterisks follow increasing *p*-values: *p* < 0.05 for *; *p* < 0.01 for **; *p* < 0.001 for ***.

	Diseased (D)	Global (G)	H Floor	H Understory	H Canopy	D Floor	D Understory	D Canopy	G Floor	G Understory	G Canopy
Healthy (H)	0.51 *	0.61 *	0.68 *	0.82 **	0.58 *	0.58 *		0.54 *	0.61 *		
Diseased (D)	—	0.84 ***		0.77 **		0.84 ***		0.72 **		0.59 *	0.59 *
Global (G)		—		0.72 **		0.84 **		0.75 **	0.62 *		0.73 **
H Floor			—						0.68 *		
H Understory				—		0.76 **		0.64 *			
H Canopy					—			0.67 *			0.64 *
D Floor						—			0.72 **	0.50 *	
D Understory							—			0.82 ***	
D Canopy								—			0.93 ***
G Floor									—		
G Understory										—	
G Canopy											—

## Data Availability

Data are available on request from the corresponding author.
